# Consumer Participation in Quality Improvements for Chronic Disease Care: Development and Evaluation of an Interactive Patient-Centered Survey to Identify Preferred Service Initiatives

**DOI:** 10.2196/jmir.3545

**Published:** 2014-12-19

**Authors:** Elizabeth A Fradgley, Christine L Paul, Jamie Bryant, Ian A Roos, Frans A Henskens, David J Paul

**Affiliations:** ^1^Priority Research Centre for Health BehaviourSchool of Medicine and Public Health & Hunter Medical Research InstituteUniversity of NewcastleCallaghanAustralia; ^2^Youth Research CenterUniversity of MelbourneMelbourneAustralia; ^3^Distributed Computing Research GroupSchool of Electrical Engineering and Computer ScienceUniversity of NewcastleCallaghanAustralia

**Keywords:** ambulatory care, health care surveys, patient-centered care, consumer participation, medical oncology, chronic disease, cardiology, neurology

## Abstract

**Background:**

With increasing attention given to the quality of chronic disease care, a measurement approach that empowers consumers to participate in improving quality of care and enables health services to systematically introduce patient-centered initiatives is needed. A Web-based survey with complex adaptive questioning and interactive survey items would allow consumers to easily identify and prioritize detailed service initiatives.

**Objective:**

The aim was to develop and test a Web-based survey capable of identifying and prioritizing patient-centered initiatives in chronic disease outpatient services. Testing included (1) test-retest reliability, (2) patient-perceived acceptability of the survey content and delivery mode, and (3) average completion time, completion rates, and Flesch-Kincaid reading score.

**Methods:**

In Phase I, the Web-based Consumer Preferences Survey was developed based on a structured literature review and iterative feedback from expert groups of service providers and consumers. The touchscreen survey contained 23 general initiatives, 110 specific initiatives available through adaptive questioning, and a relative prioritization exercise. In Phase II, a pilot study was conducted within 4 outpatient clinics to evaluate the reliability properties, patient-perceived acceptability, and feasibility of the survey. Eligible participants were approached to complete the survey while waiting for an appointment or receiving intravenous therapy. The age and gender of nonconsenters was estimated to ascertain consent bias. Participants with a subsequent appointment within 14 days were asked to complete the survey for a second time.

**Results:**

A total of 741 of 1042 individuals consented to participate (71.11% consent), 529 of 741 completed all survey content (78.9% completion), and 39 of 68 completed the test-retest component. Substantial or moderate reliability (Cohen’s kappa>0.4) was reported for 16 of 20 general initiatives with observed percentage agreement ranging from 82.1%-100.0%. The majority of participants indicated the Web-based survey was easy to complete (97.9%, 531/543) and comprehensive (93.1%, 505/543). Participants also reported the interactive relative prioritization exercise was easy to complete (97.0%, 189/195) and helped them to decide which initiatives were of most importance (84.6%, 165/195). Average completion time was 8.54 minutes (SD 3.91) and the Flesch-Kincaid reading level was 6.8. Overall, 84.6% (447/529) of participants indicated a willingness to complete a similar survey again.

**Conclusions:**

The Web-based Consumer Preferences Survey is sufficiently reliable and highly acceptable to patients. Based on completion times and reading level, this tool could be integrated in routine clinical practice and allows consumers to easily participate in quality evaluation. Results provide a comprehensive list of patient-prioritized initiatives for patients with major chronic conditions and delivers practice-ready evidence to guide improvements in patient-centered care.

## Introduction

### Background

In the past decade, chronic diseases have become the leading cause of death worldwide and are associated with 59% of deaths and 46% of the global disease burden [1]. Prevalent chronic diseases include hypertension, diabetes mellitus, arthritis, asthma, chronic obstructive pulmonary disease, nonmelanoma cancers, and depression [2,3]. Care for chronic diseases usually requires comprehensive, personalized, and long-term services involving multidisciplinary teams. This complex care is often delivered by outpatient clinics, which are defined as services providing diagnostic or therapeutic care not requiring an overnight stay in a medical institution [4].

Within most developed countries, hospital-based outpatient clinics provide a substantial proportion of health care services and require considerable resources. For example, within Australia, hospital outpatient costs in 2011 represented approximately 61% of all health care spending [5]. The National Hospital Ambulatory Medical Care Survey reported 96.1 million outpatient department visits within the United States in 2009 alone [6]. Therefore, quality assurance initiatives targeting hospital-based outpatient services have the potential to deliver substantial benefits from both a patient perspective and a health service efficiency perspective.

A patient-centered framework is a critical component to improving chronic disease care. Patient-centered care recognizes the values, preferences, and involvement of patients and their loved ones and establishes patients as an expert information source for assessing health care quality [7]. This quality indicator has been adopted into both evaluation practice and national policies including the Australian National Health Performance Framework [8]; the United Kingdom’s National Standards, Local Action, Health and Social Care Standards and Planning Framework [9]; and the Canadian Institute for Health Information’s Health Indicators [10].

Appropriate measurement of patient-centered care is essential to quality evaluation practices. Patient satisfaction surveys and unmet need measures, such as the Supportive Care Need Survey [11] and the Camberwell Assessment of Needs [12], elicit participants’ evaluations of outpatient care and are traditionally administered in pen-and-paper format [13]. These tools allow consumers to identify existing gaps in care and summarize perceptions of health services. For example, studies of cancer patients indicated that most were satisfied with their overall care [14], but improvement was needed regarding information, relationship, and patient care needs [15-17]. Outpatients with mental disorders reported unmet needs in psychological, relationship, and activities of daily living domains [18]. Other groups, such as patients with cardiovascular disease, reported unmet information and psychological needs [19]. Overall, such literature suggests that health care services struggle to address the needs of patients who require frequent care and have greater disease severity [20]. Results from intervention studies also suggest that attempts to translate results from needs assessment tools into practice has limited or inconsistent effects on care, outcomes, and satisfaction [13,21,22].

### Practice-Ready Evidence and Consumer Engagement in Designing Health Service Initiatives

The gap in translating unmet needs to improved patient-centered care may be related to difficulties in operationalizing the results of needs assessment tools. To operationalize these data and influence practice, it is important to gather additional evidence to identify patients’ preferences for changes within their health care services, strategically introduce initiatives according to patients’ priorities, provide clear and feasible service-level targets for initiatives, and provide sufficient detail to design initiatives that align closely with patients’ preferences and priorities. Static needs assessment tools are generally not designed to deliver such comprehensive, practice-ready, and influential data across multiple chronic conditions.

First, needs assessment tools do not enable patients to be highly specific about which unmet needs should be addressed within outpatient clinics. For example, although existing tools may facilitate patients’ identification of loneliness as an unmet need, patients may not expect health professionals to provide support for this issue [13]. For unmet needs that patients do want addressed within outpatient clinics, the level of detail provided by current needs assessment tools is unlikely to be adequate. For example, parking is a frequently identified unmet need, but it is reported without specificity regarding what could be changed—spaces for clinic patients only or drop-off zones for caregivers? Without a tool capable of identifying which specific action is most likely to improve patients’ experiences, health services may fail to resolve the issue. A Web-based tool with adaptive questioning would allow participants to provide information that is more detailed and personally relevant and would eliminate the time and effort required to navigate inapplicable content.

Second, an accurate method of identifying initiatives in order of priority is needed to direct limited health resources to those of greatest importance to patients. In a recent literature review of needs assessment tools frequently used in oncology care, no tool included a priority setting exercise capable of generating a comprehensive yet concise list of specific service initiatives [23]. Efforts that generate such information, such as willingness to pay or contingent valuation, are complex tasks for participants to complete using the traditional pen-and-paper format [24]. A Web-based tool with interactive survey content can be used to efficiently examine consumers’ priorities.

Third, to elicit change, tools must produce results in a form that can be readily used by health service providers and managers. Items identified by patients must be modifiable on a service level and recognized as relevant by the service providers who receive the information [25].

Finally, previous research suggests that to integrate patient-reported surveys into routine clinic practice, tools should be psychometrically robust, acceptable to patients with structured and comprehensive content, and feasible to administer in health care settings as measured by completion times and ease of administration [13]. Web-based survey software can be used to ensure these criteria are met. For example, research indicates use of this technology allows for improved readability and comprehension with simplified question formats, convenient data entry, reduction of missing data, complete timing statistics recorded by the software, and reduced administration times as compared to pen-and-paper versions [13,26,27].

### Need for a Comprehensive Tool to Inform Health Service Initiatives Based on Consumers’ Preferences

This study reports the development and evaluation process for an interactive Web-based tool capable of providing practice-ready, influential information suitable for designing patient-centered service initiatives for chronic disease care. This information-generating tool, the Consumer Preferences Survey, includes a set of general initiatives. Using adaptive questioning and interactive survey content, the survey also contains a comprehensive list of initiatives that are more detailed and a priority setting exercise. This will offer an alternative and efficient data collection method for identifying and introducing strategic changes to outpatient services.

This study aimed to:

1. Systematically construct a tool that (1) includes a comprehensive set of patient-centered initiatives that can be introduced at a service level, (2) allows participants to easily generate a personalized list of initiatives that would improve their experiences as an outpatient, and (3) generates practice-ready and actionable evidence in the form of a list of patient-prioritized initiatives (Phase 1).
2. Establish the following in relation to the this tool: (1) test-retest reliability, (2) patient-perceived acceptability of the survey content and delivery mode, and (3) average completion time, completion rates, and Flesch-Kincaid reading score (Phase 2).

## Methods

### Phase 1: Systematic Development and Pretesting of the Consumer Preferences Survey

#### Structured Literature and Stakeholder Review

Given the extensive qualitative work underpinning measures of need and satisfaction with patient-centered care [23,28], a literature-based approach was used to generate a comprehensive pool of item content (overarching domains and health service initiatives), preference eliciting techniques (item stems and response scales), and possible prioritization exercises.

A total of 336 articles were reviewed for item content and techniques. A total of 179 unique items and 6 unique domains were identified. Approximately 5 unique item stems were identified which incorporated concepts such as satisfaction with care, impact or value of an initiative, and perceived importance of an initiative. Four relative prioritization exercises were developed and included: ranking processes, modified willingness-to-pay questions, visual apportioning of a pie chart to respective health service initiatives, and a visual analog scale in which participants were asked to place initiatives according to importance.

Items and techniques generated by the structured literature review were circulated to 2 expert committees using an iterative process. The first committee included chronic disease physicians, health service managers from hospital-based specialist services, community-based chronic disease experts, and health behavior researchers including an academic biostatistician and a health economist (n=20). The second committee included consumer advocates and health service users within cancer, neurology, and cardiology fields (n=27).

#### Final Survey Content

**Figure 1:**
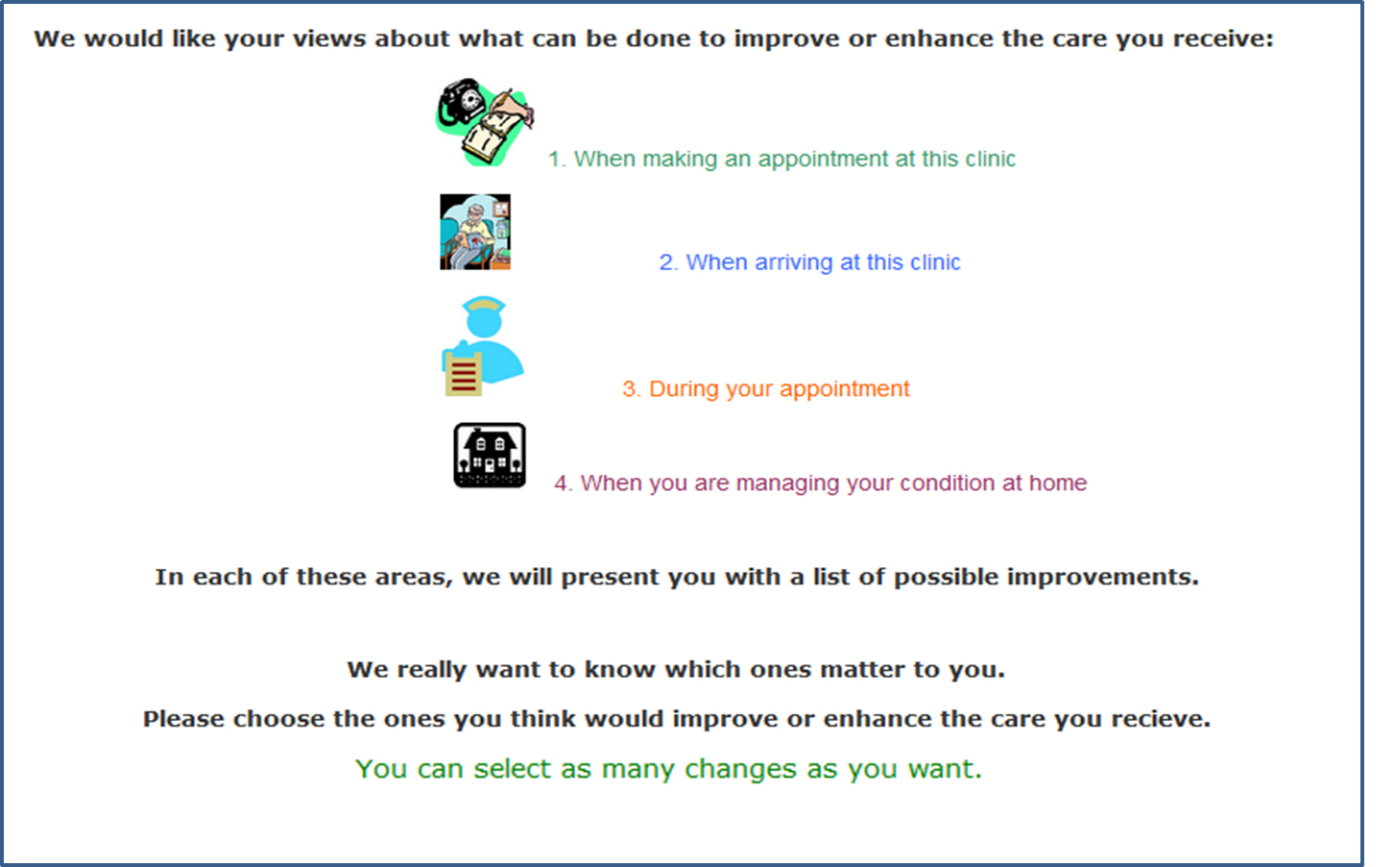
Screenshot of the Consumer Preferences Survey, introduction and instruction screen.

After 2 rounds of feedback from each expert group, a total of 23 general service initiatives were generated from the item pool (Table 1). These initiatives were organized as 4 steps in the process of care: (1) scheduling an appointment, (2) arriving at an appointment, (3) during a clinical appointment, and (4) managing a chronic illness at home (Figure 1). Both expert committees preferred this approach. By allowing participants to sequentially consider each way in which care was experienced and could be improved, recall bias and cognitive burden may be reduced. However, these areas are not considered latent variables or constructs.

**Figure 2:**
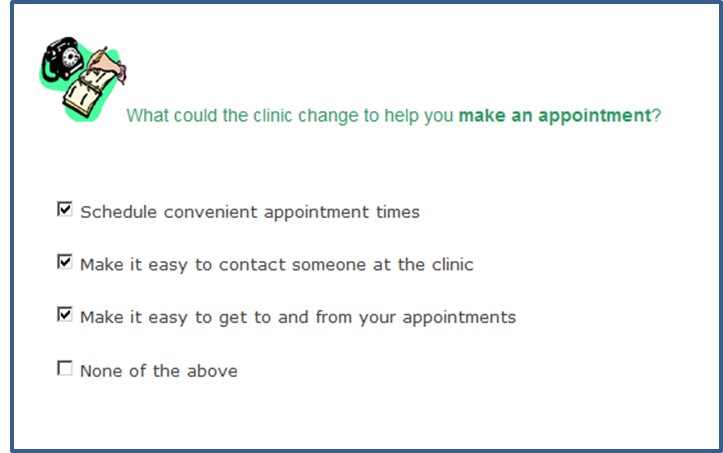
Screenshot of the Consumer Preferences Survey, selecting general initiatives.

**Figure 3:**
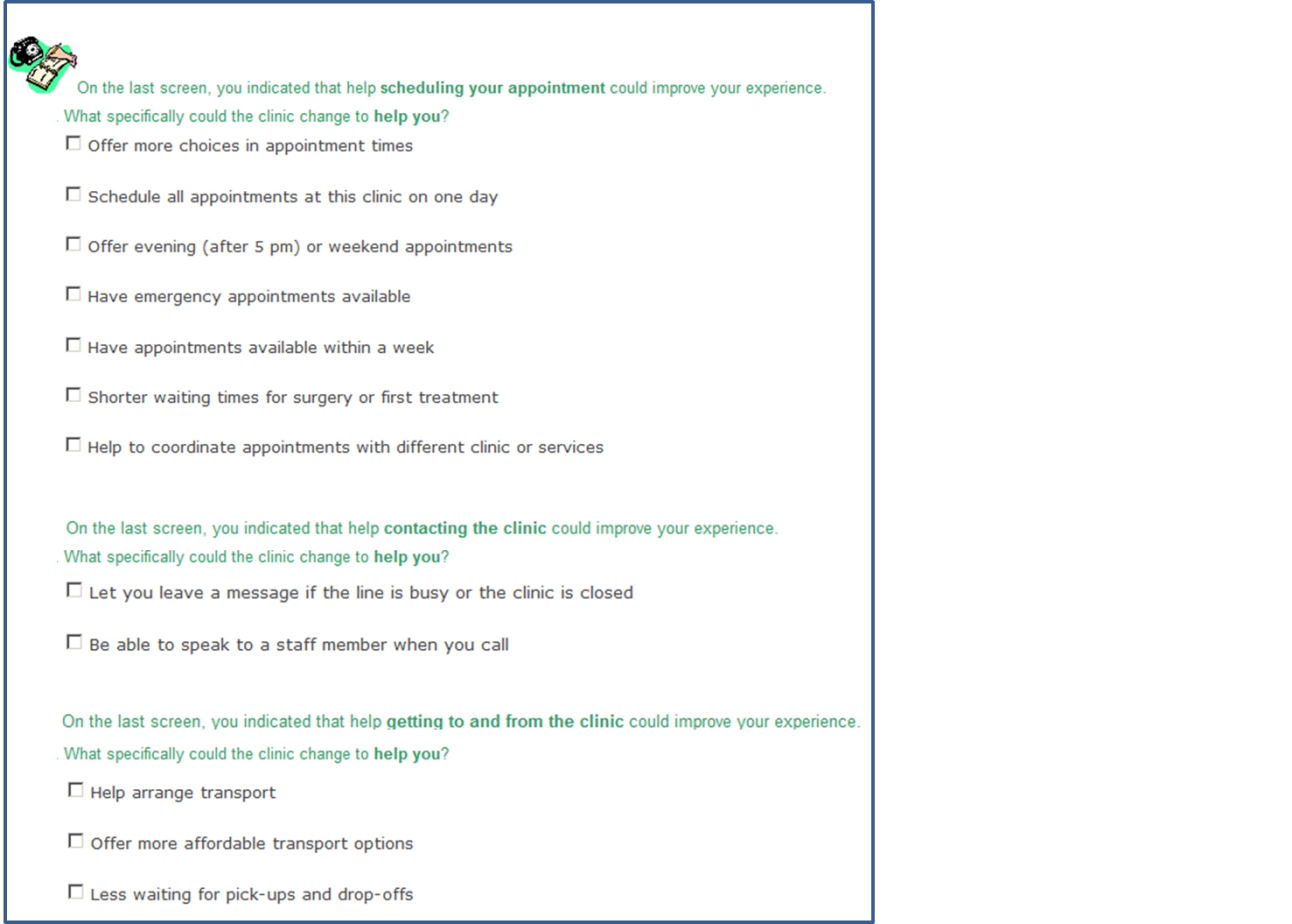
Screenshot of the Consumer Preferences Survey, selecting specific initiatives.

Participants selected initiatives that would greatly improve their experience within the outpatient clinic from lists presented on the touchscreen computer. This is considered a dichotomous response scale. Selected initiatives were recorded as 1 and unselected initiatives were coded as zero. If a general initiative was selected, the survey displayed a subsequent list of specific health service initiatives using adaptive questioning: “On the last screen, you indicated that [general health service initiative] could improve your experience (Figures 2 and 3. What specifically could the clinic change to help you? [list of possible specific health services initiatives displayed].” A total 110 specific health service initiatives were available, including having emergency appointments available within a week (making an appointment), being informed of estimated wait times on arrival (arriving at an appointment), an action plan is created to address your concerns (during an appointment), and knowing which symptoms require emergency attention (self-management at home). Only those participants who selected all 23 broad health service initiatives would view all 110 detailed health service initiatives. Complete survey content is available in Multimedia Appendix 1.

**Figure 4:**
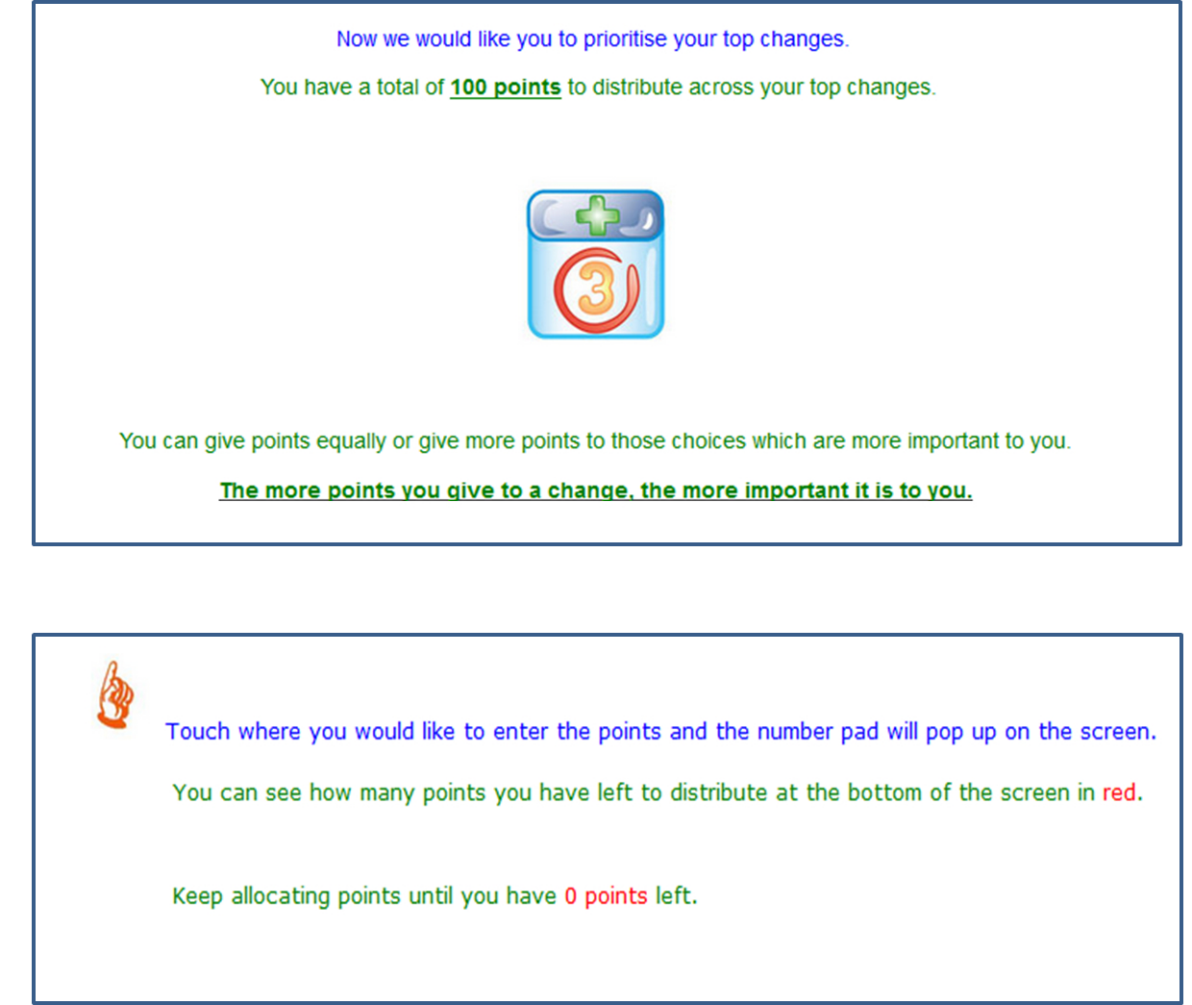
Screenshot of Consumer Preferences Survey, instructions for relative prioritization exercise.

**Figure 5:**
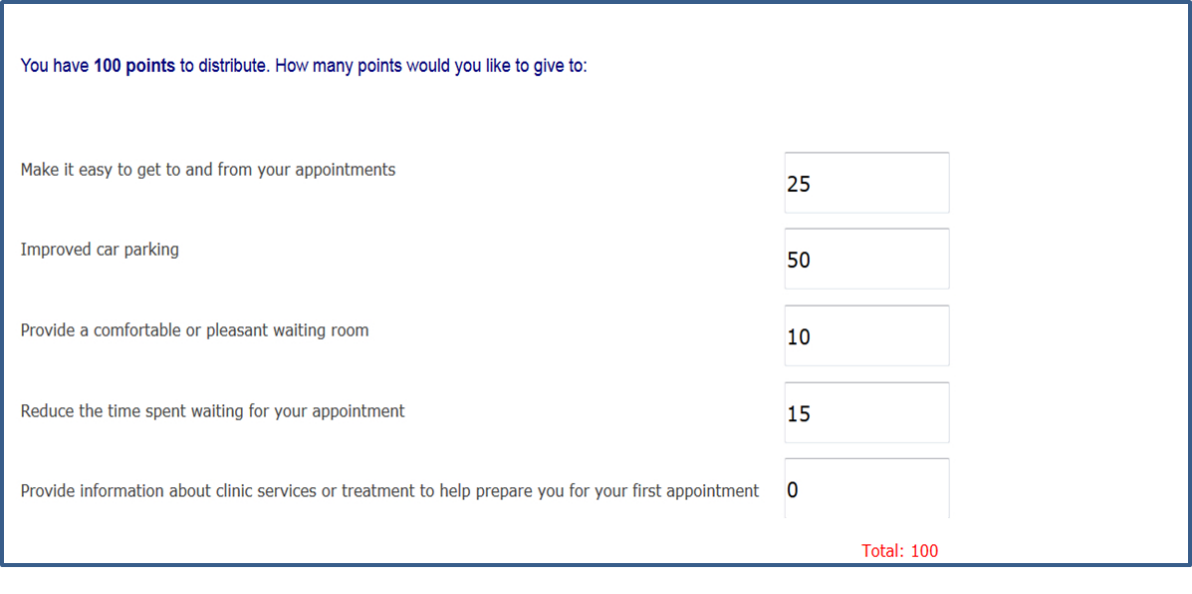
Screenshot of Consumer Preferences Survey, relative prioritization exercise.

Once the 4 steps were completed, participants who selected more than 5 general initiatives were presented with an autopopulated list of their previous selections and were asked to choose the 5 initiatives that were of greatest importance to them. These participants and those individuals who selected at least 2 but less than 5 initiatives were directed to a modified relative prioritization exercise. Participants were asked to allocate 100 points across their desired initiatives to indicate the relative perceived importance of each (Figures 4 and 5).

**Table 1:** Consumer Preferences Survey content by area of care, including number of possible specific initiatives.

Area of care and general initiatives		Specific initiatives (n)
**1. Making an appointment**		
	Schedule convenient times	7
	Easy contact with clinic staff	2
	Transportation to appointment	3
**2. Arriving at an appointment**		
	Car parking	7
	Comfortable wait rooms	10
	Reduced time in wait rooms	3
	Having friends or family with you	—
**3. During clinical appointment**		
	Provide more information	4
	Ensure all your concerns are addressed	4
	Involve you more in treatment decisions	3
	Keep you up-to-date on the progress of your treatment and condition	3
	Ensure good interactions and relationships with all clinic staff	5
	Provide comfortable and pleasant treatment rooms	10
	Provide good quality hospital catering	—
	Better coordination of your care and information	7
	Minimize pain or discomfort when you receive treatment	4
**4. Managing at home**		
	Help with physical symptoms	6
	Help with emotional health or relationships	4
	Help with daily activities and healthy lifestyles	6
	Help with employment, finances, or insurance	5
	Information on your condition and treatment	8
	Support and involvement of family and friends	5
	Knowledge on how to handle a medical emergency	4

#### Final Web-Based Format

To facilitate adaptive questioning and the branching patterns required to navigate the survey, a novel software program using touchscreen technology was developed in collaboration with health behavior researchers and information technology experts [29]. To confirm the technical functionality and usability of the survey software, a total of 75 participants pretested the final format without error.

To commence the survey, a research assistant first registered the user with a unique username before handing the touchscreen device to the participant. This registration step allowed participants to pause and restart the survey without losing previously entered information by re-entering their username. The unique username was stored with each result set, and removed before analysis, to allow detection of nonunique participants. If a duplicate was discovered, the entry with complete data only was used for analysis.

Once a username was created, participants were able to progress through the survey using a clearly presented “Next” button located at the bottom of every survey page. Participants were also able to navigate to previous responses using the “Back” button. All participants received 4 instruction screens and 4 screens listing the 23 general initiatives. In the unlikely scenario a participant selected all general initiatives, based on adaptive questioning they would receive an additional 7 screens listing 110 specific initiatives. The prioritization exercise included 2 instruction screens and 2 exercise screens. The maximum number of survey items presented on a screen was 4 and participants may have been required to page scroll to view all items.

### Phase 2: Test-Retest Reliability and Patient Acceptability of the Consumer Preferences Survey

#### Clinic Settings

High-volume tertiary outpatient medical oncology, cardiology, and neurology clinics were included to ensure the pilot sample reflected a range of prevalent chronic illnesses. Pilot sites included a public tertiary outpatient clinic specializing in both cardiology and neurology care, a public tertiary outpatient clinic specializing in oncology care, and a private tertiary outpatient clinic specializing in intravenous chemotherapy only.

#### Participant Eligibility

Eligible participants were able to read English, 18 years of age or older, and had attended the clinic at least once prior to recruitment. A subsample of participants completed the survey again within 14 days to assess test-retest reliability. Eligibility for the test-retest component of the study required written consent and a second appointment scheduled within 10-14 days. Given this narrow timeframe, only medical oncology patients with an ongoing treatment schedule were approached to participate in the test-retest component of the study.

#### Recruitment and Survey Administration

Trained research assistants approached patients in the outpatient clinic waiting rooms or intravenous chemotherapy treatment spaces. Eligible participants were invited to complete the survey at the time of recruitment only and individuals were not provided the website address to access the survey outside of the clinic setting. The survey was voluntary, not advertised, and no incentives to participate were offered. The age and gender of nonconsenters was estimated to ascertain consent bias.

#### Measures

The touchscreen survey consisted of the Consumer Preferences Survey and the following:

1. Demographic information: age, gender, marital status, education, private health insurance, concessional card, Aboriginal or Torres Strait Islander origin, and appointment frequency within the past 3 months were collected. Participants also reported the reason for attending the clinic with response options of a routine exam for a diagnosed condition, discussion of symptoms for a diagnosed or nondiagnosed condition, or to receive tests or treatments.
2. Acceptability items: a total of 6 questions assessed the acceptability of the Consumer Preferences Survey: (1) Do you think the directions given for filling out the survey were adequate?, (2) Overall, how would you rate the length of the survey? with response options “it was too short,” “it was just right,” or “it was too long,” (3) Did the survey miss any changes that could improve your experience in this outpatient clinic?, (4) Did you find filling in the survey confusing or difficult?, (5) Would you be willing to complete a similar survey in the future?, and (6) Do you believe the survey will provide an accurate summary of initiatives which could improve your experience within the outpatient clinic? Only those participants who reported difficulty completing the survey were asked additional questions assessing the ease of navigation, layout or functioning of iPad screens, adequacy of directions, and whether some changes would be helpful. Participants who selected at least 2 general initiatives and were instructed to complete the relative prioritization exercise received 3 additional questions, including (1) Do you think the directions given for this exercise were adequate?, (2) Did this exercise help you to decide which changes to the clinic are most important to you?, and (3) Did you find this exercise difficult?

#### Data Analysis

To examine test-retest reliability, nonweighted Cohen’s kappa coefficients and percent agreements were calculated to report differences between responses at participants’ first completion of the survey and second completion of the survey. Items with a kappa value equal to or greater than .60 were considered to have substantial test-retest reliability [30]. Those items reporting a kappa value from .40 to .59 were considered to have moderate test-retest reliability.

Acceptability items were examined using proportions and 95% confidence intervals. Differences in estimated age, gender, or clinic characteristics of consenters or nonconsenters were examined using chi-square statistics. A *P* value of <.05 was considered statistically significant.

Ease of integration of the Consumer Preferences Survey was assessed by examining Flesch-Kincaid reading level [31], average time to complete, and survey completion rates. The survey software recorded timing statistics and survey completion rates. The average time to complete, including standard deviations, each portion of the survey is reported.

#### Institutional Review Board Approval and Data Protection

Ethics approval was provided by Hunter New England Human Research Ethics Committee and the University of Newcastle Human Research Ethics Committee. Consent was implied if an individual chose to begin the survey. All personal information was immediately uploaded via an encrypted channel and stored on secure university servers with password-protected access granted to study researchers only.

## Results

### Summary

A total of 1042 chronic disease outpatients were approached to participate over a 10-month period from March to December 2013. A total of 741 individuals agreed to participate—a 71.11% consent rate (Table 2). Of the 301 individuals who declined to participate, clinic site was documented and age and gender estimated for 291 individuals (96.7%). Of the 741 consenting participants, age, gender, and clinic sites were recorded for 674 individuals (91.0%). There were no significant differences between consenters and nonconsenters by gender (*P*=.85). Age category was significantly related to consent (*P*=.007). Consent rates were also significantly higher within the privately funded intravenous chemotherapy clinic compared with both the publically funded oncology clinic and publically funded cardiology and neurology clinic (*P*=.001).

A total of 143 of 184 medical oncology participants (consent rate 78.1%) were willing to participate in the test-retest component. Only 68 of these 143 had a scheduled appointment within 14 days (48.9% eligibility). Due to rescheduled appointments and participants’ physical well-being at the second appointment, 39 participants were included in the final test-retest sample.

**Table 2:** Demographic characteristics by consent status for Consumer Preferences Survey pilot (N=965).

Demographic characteristic		Nonconsenters, n (%) (n=291)	Consenters, n (%) (n=674)	χ^2^ (*df*)	*P*
Male (n=438)		134 (46.2)	304 (45.1)	0.04 (1)	.85
**Clinic site**				14.7 (2)	.001
	Public oncology (n=476)	148 (31.1)	328 (68.9)		
	Public cardiology and neurology (n=415)	135 (32.5)	280 (67.5)		
	Private oncology (n=74)	8 (10.8)	66 (89.2)		
**Age category**				14.1 (4)	.007
	18-25 (n=38)	5 (2.7)	33 (4.6)		
	26-40 (n=155)	49 (16.8)	106 (16.1)		
	41-55 (n=262)	80 (27.9)	182 (25.4)		
	56-70 (n=354)	95 (31.9)	259 (35.8)		
	≥71 (n=156)	62 (20.8)	94 (18.2)		

### Sample Demographic Characteristics

A total sample of 674 participants completed the demographic module and included 394 medical oncology patients (58.5%) and 280 (41.5%) cardiology or neurology patients (Table 3). Females were slightly overrepresented (54.9%, 370/674) in the sample and the average age was approximately 59.7 years (SD 15.5 years). Participants were most likely to have attained a high school equivalent of year 10 or lower (49.2%, 332/674) and to be married or living with a partner (66.3%, 447/674). The most common reported reasons for attending the clinic were related to a diagnosed condition, with 41.9% (282/674) of participants attending for a routine exam and 30.5% (206/674) attending to receive tests or treatment.

**Table 3:** Sample demographic characteristics of Consumer Preferences Survey pilot test (N=674).

Sample characteristics		Participants
**Age (years), mean (SD)**		59.7 (15.5)
**Male, n (%)**		304 (45.1)
Highest level of education attained, n (%)		
	High school equivalent of year 10 or lower	332 (49.2)
	High school completion	93 (13.8)
	Diploma or trade certificate	140 (20.8)
	Bachelor’s degree	63 (9.4)
	Not specified	46 (6.8)
Marital status, n (%)		
	Married or living with partner	447 (66.3)
	Single	84 (12.5)
	Widowed	84 (12.5)
	Not specified	59 (8.7)
**Aboriginal and/or Torres Strait Islander origin, n (%)**		28 (4.2)
**No private insurance coverage, n (%)**		361 (53.6)
**Concessional card, n (%)**		448 (66.5)
Chronic condition group, n (%)		
	Cardiology or neurology	280 (41.5)
	Medical oncology	394 (58.5)
**Medical oncology private facility, n (%)**		66 (9.8)
Reason for attending, n (%)		
	To discuss symptoms, treatments or tests for diagnosed condition	121 (17.9)
	To discuss symptoms or tests for undiagnosed condition	49 (7.3)
	To receive tests or treatments for diagnosed condition	206 (30.5)
	For a routine exam for a diagnosed condition	282 (41.9)
	Not specified	16 (2.4)
Number of appointments in last 3 months, n (%)		
	1 in last 6 months	328 (48.7)
	2-3	145 (21.5)
	4-5	92 (13.6)
	6	31 (4.6)
	≥7	73 (10.8)
	Not specified	3 (0.8)

### Reliability Statistics

A total of 39 oncology patients participated in the test-retest component (Table 4). Substantial test-retest reliability was reported for 9 general initiative items (Cohen’s kappa>.6) and moderate test-retest reliability was reported for 7 general initiatives (Cohen’s kappa=.40-.59). Four initiatives reported a value below a .4 threshold, indicating poor reliability. However, observed agreement for these items ranged from 94.9%-97.4%. There were an insufficient number of observations to calculate a test statistic for 3 initiatives. Across all initiatives, the average observed agreement was 93.7% with moderate test-retest reliability (Cohen’s kappa=.53).

**Table 4:** Cohen’s kappa values and observed percentage agreement for general initiatives (n=39).

General initiatives selected by area of care		Observed agreement (%)	Cohen’s κ (95% CI)
**1. Area of care: making an appointment**			
	Provide more convenient appointment times	89.7	.44 (–.01, .09)
	Make it easier to contact the clinic	94.9	.64 (.18, 1.00)
	Help to arrange transport to and from the clinic	94.9	.47 (.15, 1.00)
	None selected	89.7	.69 (.40, .97)
	Total number selected	87.2	.62 (.46, .68)
**2. Area of care: arriving at an appointment**			
	Improve car parking	89.7	.79 (.59, .98)
	Provide a comfortable and pleasant waiting room^a^	—	—
	Reduce waiting times	94.9	.47 (–.15, 1.00)
	Ensure family and friends are comfortable within waiting rooms	97.4	.66 (.03, 1.00)
	None selected	82.1	.64 (.40, .88)
	Total number selected	84.6	.71 (.52, .95)
**3. Area of care: arriving at an appointment**			
	Provide more information about treatment and condition	97.4	0
	Ensure your concerns are discussed with health care professionals	97.4	.79 (.38, 1.00)
	Involve you in treatment decisions	100.0	1.00 (1.00, 1.00)
	Keep you up-to-date on your treatment and condition progress	92.3	.53 (.06, .99)
	Ensure good interactions with all clinic staff	97.4	0
	Provide a comfortable and pleasant treatment room^a^	—	—
	Provide good hospital catering	89.7	.55 (.18, .92)
	Better coordination of your care	97.4	.66 (.03, 1.00)
	Minimize pain or discomfort during treatment^a^	-	—
	None selected	84.6	.60 (.32, .88)
	Total number selected	79.5	.50 (.33, .80)
**4. Area of care: managing your condition at home**			
	Access to help or information to manage physical symptoms	97.4	.66 (.03, 1.00)
	Access to help or information to manage emotional symptoms	94.9	–.03 (–.09, .04)
	Access to help in order to maintain activities of daily living	94.9	–.03 (–.09, .04)
	Access to help or information relating to finance, work, insurance	97.4	.84 (.54, 1.00)
	Access to information to review at home	94.9	.48 (–.12, 1.00)
	Access to help or information for family support	94.9	.48 (–.12, 1.00)
	Information on how to manage medical emergencies	94.9	.64 (.19, 1.00)
	None selected	89.7	.72 (.46, .97)
	Total number selected	84.6	.60 (.29, .63)

^a^ Insufficient number of observations to calculate a test statistic.

### Acceptability Statistics

A total of 543 of 674 individuals (80.6%) completed the acceptability items related to selecting and navigating general initiatives and 529 (78.9%) completed items related to the perceived value of survey results (Table 5). This noncompletion rate was observed for those participants who were called into their appointment before completing the survey. Of the 543 participants, most found the Consumer Preferences Survey easy to complete (97.9%, 531/543), comprehensive (93.1%, 506/543), an appropriate length (95.5%, 519/543), and thought adequate directions were provided (98.3%, 534/543). Of the 195 participants who received the relative prioritization exercise, the majority indicated it was easy to complete (97.0%, 189/195) and that directions were clear (94.6%, 184/195).

A minority of participants indicated they were unsure if the results were an accurate summary of the initiatives desired (17.4%, 92/529) or were unsure if they would be willing to complete a similar survey in future (9.9%, 52/529). However, the majority of participants (80.7%, 427/529) believed the survey results were an accurate summary of initiatives that could improve their experience within the outpatient clinic, whereas 84.6% (165/195) reported the relative prioritization exercise helped them to decide which general initiatives were of greatest importance. Overall, 84.6% (448/529) of participants were willing to complete a similar survey in the future. The Flesch-Kincaid reading level was grade 6.8, indicating that those who completed 7 years of formal education would be able to easily comprehend the survey content.

**Table 5:** Reported acceptability of the Consumer Preferences Survey, including relative prioritization exercise.

Acceptability		Participants, % (95% CI)
Selecting and navigating general initiatives (n=543)		
	The directions provided were adequate	98.3 (97.2-99.6)
	The length of the survey was appropriate	95.5 (93.5-97.5)
	The survey was comprehensive of all initiatives	93.1 (90.7-95.6)
	The survey was clear and easy to complete	97.9 (96.4-99.3)
Completing the relative prioritization exercise^a^ (n=195)		
	The directions for the points exercise was adequate	94.6 (92.2-97.0)
	The point exercise helped to decide what was important	84.6 (80.4-88.8)
	The point exercise was clear and easy to complete	97.0 (95.2-98.8)
Overall value of survey (n=529)		
	The survey is an accurate summary of the initiatives desired	80.7 (77.0-84.5)
	Willing to complete a similar survey in the future	84.6 (81.0-88.2)

^a^ Completed by only those participants with 2 or more general initiatives selected.

### Completion Rates and Timing Statistics

Approximately 78.4% (529/674) of participants completed the Consumer Preferences Survey and all acceptability questions. Completion was significantly related to clinic site (data not shown; *P*<.001), with completion rates significantly higher within the privately funded intravenous chemotherapy clinic (98.5%, 65/66) compared to the publically funded oncology clinic (82.3%, 325/394). Both oncology clinics reported significantly higher completion than publically funded cardiology and neurology clinic (69.3%, 194/280).

Approximately 5 minutes (mean 5.02, SD 3.07) was required to navigate and select initiatives and to complete the relative prioritization exercise. The total time to complete all pilot survey content, excluding acceptability questions, was approximately 8.54 (SD 3.91) minutes.

## Discussion

### Principal Results

The development of the Consumer Preferences Survey was successful in providing a novel tool capable of generating a personalized and concise list of health service initiatives relevant to patients’ experiences of outpatient care, identifying a comprehensive set of targets that are modifiable on a service level, and generating a list of prioritized initiatives to ensure service-level change is introduced strategically. The interactive survey software also allows participants to select up to 110 specific initiatives and indicate the relative importance of chosen initiatives in improving their care experience.

Results from our pilot study suggest the tool is sufficiently reliable and acceptable to patients. The test-retest reliability of each general initiative was moderate to substantial and observed percentage agreement was above 80%, indicating that this tool provides a stable summary of patients’ preferences for health service change. Participants reported the Consumer Preferences Survey was easy to complete, comprehensive, and of an appropriate length. Based on average completion times and reading level, this tool can also be integrated into routine clinic practice and allows consumers to quickly participate in a quality evaluation exercise. Time to complete is approximately 9 minutes and is comparable to, or shorter than, other patient-report tools, such as the Cancer Care Monitor (12 minutes), Supportive Care Needs Survey (15-20 minutes), and Camberwell Assessment of Need (16 minutes) [12,13]. The Flesch-Kincaid reading level of the survey was assessed at 6.8. This requires participants to have completed a level of formal education well below the level of education recommended by the Australian National Health Medical Research Council for presenting information to health consumers [32].

Electronic touchscreen surveys are becoming a popular mode of survey administration within health research [33]. The Consumer Preferences Survey uses innovative Web-based software capable of complex adaptive questioning and interactive item types. The branching patterns allow participants to easily navigate through all general initiatives and only receive subsequent questions focusing on specific initiatives when appropriate. The survey content, particularly the way in which participants receive questions and the relative prioritization exercise, is a novel approach to summarizing and prioritizing patients’ perceptions of the quality of care. As such, it is promising that approximately 85% of individuals indicated they would be willing to complete a similar survey in the future and only 2% believed the survey did not provide an accurate summary of desired health service initiatives. The relative prioritization exercise was perceived to be a helpful and easy exercise that could be completed in a relatively short amount of time. Similar Web-based exercises have been successfully used to explore consumers’ research priorities and decision preferences [24,34]. With limited health care resources available, simplified willingness-to-pay exercises may be an appropriate data collection approach to strategically determine funding priorities according to consumers’ preferences.

### Value and Application of the Consumer Preferences Survey

The involvement of consumers in shaping health policy and services is widely recognized as important for promoting patient-centered care in chronic diseases. Firstly, it is seen as an ethical and democratic right [35]. This can be an empowering experience for consumers who have been traditionally passive receivers of care with little opportunity to discuss their experiences. Secondly, consumers also offer a different but equally important perspective on the quality of health care than those of health service providers, researchers, and policy makers [35]. As research funding organizations and government health policies continue to mandate consumer involvement, a process to systematically collect and measure consumers’ perspectives of care is needed.

The Consumer Preferences Survey allows consumers to participate in a quality evaluation exercise and provides valuable information on how health services can be restructured. This is an information-generating tool and can be used to determine consumers’ preferences and priorities for health service initiatives. The data collection process is systematic, meaningful to consumers and health services, and sufficiently detailed and concise to translate into meaningful patient-centered health initiatives. The touchscreen survey covers a wide range of issues relatively quickly to minimize participant burden and maximize the feasibility of administering the tool in a range of health care settings.

### Limitations of the Consumer Preference Survey

Unlike tools such as the Patient Generated Index [36], the Consumer Preferences Survey does not allow participants to nominate other aspects of their care they would like changed. However, these tools are not amenable to touchscreen mode of administration and cannot incorporate benefits such as an interactive action-prioritization exercise. Furthermore, a format allowing participants to generate new (and potentially highly personalized) items introduces challenges in relation to the prioritization of initiatives across chronic condition groups and users. As part of the evaluation process, the pilot test allowed participants to suggest additional initiatives that had not been covered by the survey. Only 5% of individuals suggested an additional initiative. Suggested initiatives were often specific initiatives that had been eliminated by expert review because they were deemed nonmodifiable or relevant to only a very small portion of service users.

The Consumer Preferences Survey has not been tested for validity following some traditional psychometric methods, such as factor analysis, given the lack of common denominator for items due to use of a sophisticated branching pattern. Furthermore, the organization structure of the survey into 4 distinct chronological areas of care eliminates the possibility of item randomization. These areas of care serve only as an organization structure and do not infer latent variables or construct dimensions. However, given that the Consumer Preference Survey was not designed to measure a psychological construct or diagnose a disease state, but rather as a tool for identifying and prioritizing subjective changes to hospital-based outpatient care, reliability is likely to be the most appropriate and important psychometric characteristic to establish. To further establish the credibility of survey results, additional research replicating the reliability portion of this pilot study with a greater sample size is required.

Although this pilot study included a range of settings and a large number of participants, there are several methodological limitations that may introduce bias. Firstly, age and gender were not recorded for all consenting individuals (9% missing data). As described, participants were recruited in clinic waiting rooms before their appointment or in treatment rooms while receiving intravenous therapy. All participants recruited within the privately funded clinic completed the survey while receiving intravenous treatment and did not experience time constraints. Missing data are observed for those participants called into their appointment before survey completion; this predominately occurred within the publically funded clinics. This difference in recruitment location explains the significant difference between clinic site and completion rates. There are a number of benefits that justify applying an active recruitment method within the health service settings. Firstly, participants can use the touchscreen device and survey software specifically designed for this study instead of more laborious methods, such as a postal survey or arranging a telephone interview. Secondly, participation rates are much higher with face-to-face interaction and may mitigate any response bias [37]. This can result in a more equitable representation of patients’ preferred initiatives. Although it could be argued that this approach introduces social desirability bias, this may be mitigated by the touchscreen technology which prior research suggests is a very private and acceptable data collection method [38].

Within the pilot, age was significantly related to consent. Individuals aged 71 years or older were more likely to decline participation than any other age group. There is evidence suggesting that age is generally not a barrier to using touchscreen technology within ambulatory settings [33]; within the study, the second oldest age group (50-69 years) reported a slightly higher than average consent rate (73%). This may suggest this result is isolated or due to an additional explanatory variable, such as increasing illness severity within the older age group. However, this cannot be confirmed within the existing dataset and requires further evaluation.

### Conclusions

Results from the Consumer Preferences Survey can be used to guide patient-centered care initiatives within health services and will provide a list of patient-prioritized targets across several chronic conditions. This will offer an alternative and reliable method to introduce strategic initiatives to chronic disease outpatient services with the objective of empowering consumers to participate in quality improvement activities.

## Multimedia Appendix 1

Screenshots of the Consumer Preferences Survey.
