# Supplementary material for: Consumer Participation in Quality Improvements for Chronic Disease Care: Development and Evaluation of an Interactive Patient-Centered Survey to Identify Preferred Service Initiatives
Source: J Med Internet Res. 2014 Dec 19;16(12):e292. doi: 10.2196/jmir.3545 (PMC4285719; doi:10.2196/jmir.3545)
Supplement: Supplementary file 1 [file jmir_v16i12e292_app1.pdf]

INSTRUCTION SCREEN

We would like your views about what can be done to improve or enhance the care you receive:

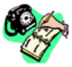
1. When making an appointment at this clinic

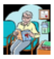
2. When arriving at this clinic

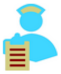
3. During your appointment

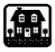
4. When you are managing your condition at home

In each of these areas, we will present you with a list of possible improvements.

We really want to know which ones matter to you.

Please choose the ones you think would improve or enhance the care you recieve.

You can select as many changes as you want.

GENERAL INITIATIVES: Making an appointment

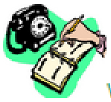
What could the clinic change to help you **make an appointment**?

- [x] Schedule convenient appointment times
- [x] Make it easy to contact someone at the clinic
- [x] Make it easy to get to and from your appointments
- [ ] None of the above

SPECIFIC INITIATIVES: Making an appointment

On the last screen, you indicated that help **scheduling your appointment** could improve your experience. What specifically could the clinic change to **help you**?

- ☐ Offer more choices in appointment times
- ☐ Schedule all appointments at this clinic on one day
- ☐ Offer evening (after 5 pm) or weekend appointments
- ☐ Have emergency appointments available
- ☐ Have appointments available within a week
- ☐ Shorter waiting times for surgery or first treatment
- ☐ Help to coordinate appointments with different clinic or services

On the last screen, you indicated that help **contacting the clinic** could improve your experience. What specifically could the clinic change to **help you**?

- ☐ Let you leave a message if the line is busy or the clinic is closed
- ☐ Be able to speak to a staff member when you call

On the last screen, you indicated that help **getting to and from the clinic** could improve your experience. What specifically could the clinic change to help you?

- ☐ Help arrange transport
- ☐ Offer more affordable transport options
- ☐ Less waiting for pick-ups and drop-offs

GENERAL INITIATIVES: Arriving at an appointment

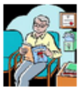

What could the clinic change to help you **before your appointment**?

- ☑ Car parking
- ☑ Comfortable or pleasant waiting room
- ☑ Reduced time in the waiting room
- ☑ Having family or friends with you in the waiting room
- ☐ None of the above

SPECIFIC INITIATIVES: Arriving an appointment

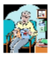

On the last screen you indicated that help with **parking** could improve your experience. What specifically could the clinic change to **help you**?

- ☐ Affordable options for parking
- ☐ Easy to use parking machines
- ☐ Spaces for clinic patients only
- ☐ Available parking close to the clinic
- ☐ More disabled only parking
- ☐ Patient drop-off zones or short stay parking for caregivers
- ☐ Providing information to patients about parking options

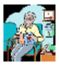

On the last screen you indicated that the clinic could **improve wait times**. What specifically could the clinic change to **help you**?

- ☐ Paging system so you can leave the waiting room, but be paged to return
- ☐ Only be required at the clinic 5 minutes before scheduled appointment
- ☐ Be informed of wait times upon arrival

SPECIFIC INITIATIVES: Arriving an appointment

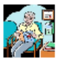

On the last screen you indicated the clinic could **improve the waiting room**. What specifically could the clinic change to **help you**?

- ☐ Paint or decorate the walls
- ☐ Improve maintenance and cleaning
- ☐ Add comfortable furniture
- ☐ Provide enough furniture for patients and family
- ☐ Reduce noise
- ☐ Better reading material, or television to occupy your time
- ☐ Wireless internet access
- ☐ Visible clock
- ☐ Privacy from people walking by (e.g. frosted glass)
- ☐ Ability to make hot or cold refreshments

GENERAL INITIATIVES: During an appointment

What could the clinic change to help you **during your appointment**?

- [x] Provide more information
- [x] Ensure all your concerns are addressed
- [x] Involve you more in treatment decisions
- [x] Keep you up-to-date on the progress of your treatment and condition
- [x] Ensure good interactions and relationships with all clinic staff
- [x] Provide comfortable and pleasant treatment rooms
- [x] Provide good quality hospital catering
- [x] Better coordination of your care and information
- [x] Minimise pain or discomfort when you receive treatment
- [ ] None of the above

SPECIFIC INITIATIVES: During an appointment

On the last screen you indicated that help **getting information** could improve your experience. What specifically could the clinic change **to help you**?

- [ ] Provide clear explanations of your treatment and condition
- [ ] Provide written information or a list of available resources to take home
- [ ] Ask at the end of each appointment if you have any questions or need additional information
- [ ] Provide personalized written information to take home

On the last screen you indicated that having **all your concerns addressed** would improve your experience. What specifically could the clinic **to help you**?

- [ ] Ensure there is enough time to ask questions and raise concerns
- [ ] Ensure a plan is made to address your concerns
- [ ] Ask at the end of each appointment if you have any other concerns
- [ ] Have a handout with common concerns or questions to review or complete with your doctor

SPECIFIC INITIATIVES: During an appointment

On the last screen you indicated that being more **involved in treatment decisions** could improve your experience. What specifically could the clinic change **to help you**?

- ☐ Provide more information on treatment options before you decide
- ☐ Provide more time to consider your options
- ☐ Your preferences and lifestyle are discussed and considered when making a decision

On the last screen you indicated that being **kept up to date on your treatment progress and condition** could improve your experience. What specifically could the clinic change **to help you**?

- ☐ Ensure you receive test results as soon as possible
- ☐ Ensure you know the status of your condition
- ☐ Ensure you are aware of the next steps in your treatment

SPECIFIC INITIATIVES: During an appointment

On the last screen you indicated that **better interactions and relations with clinic staff** could improve your experience. What specifically could the clinic change **to help you**?

- ☐ Quick and sensitive response to your needs
- ☐ Being given bad news respectfully
- ☐ Be reassured by staff how you feel is normal
- ☐ Encourage a friendly, caring environment

On the last screen you indicated that the clinic could **improve the treatment rooms**. What specifically could the clinic change **to help you**?

- ☐ Paint or decorate the walls
- ☐ Improve maintenance and cleaning
- ☐ Add comfortable furniture
- ☐ Provide enough furniture for patients and family
- ☐ Reduce noise
- ☐ Better reading material, or television to occupy your time
- ☐ Wireless internet access
- ☐ Visible clock
- ☐ Privacy from people walking by (e.g. frosted glass)
- ☐ Ability to make hot or cold refreshments

SPECIFIC INITIATIVES: During an appointment

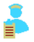

On the last screen you indicated that **help coordinating your care and information** could improve your experience. What specifically could the clinic change **to help you**?

- [ ] All clinic staff have your up-to-date information and medical history
- [ ] Healthcare providers communicate with each other
- [ ] Seeing the same healthcare providers at each appointment
- [ ] Short wait times for referrals to other healthcare providers
- [ ] Consistent information and advice from health care providers
- [ ] Having one healthcare provider to coordinate your care
- [ ] Knowing who to contact for a specific issue

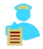

On the last screen you indicated that **minimizing pain and discomfort during treatment** could improve your experience. What specifically could the clinic change **to help you**?

- [ ] Check you are warm and comfortable while receiving treatment
- [ ] Ensure all healthcare providers are skilful at giving needles or IV lines
- [ ] Ensure immediate treatment side effects are managed
- [ ] Ensure healthcare providers monitor how you are feeling during treatment

GENERAL INITIATIVES: Self-management at home

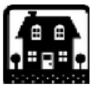

What could the clinic change to assist you with your condition **when at home**?

- [x] Help with physical symptoms
- [x] Help with emotional health or relationships
- [x] Help with daily activities and healthy lifestyles
- [x] Help with employment, finances or insurance
- [x] Information on your condition and treatment
- [x] Support and involvement of family and friends
- [x] Knowledge on how to handle a medical emergency
- [ ] None of the above

SPECIFIC INITIATIVES: At home

On that last screen you indicated that **help with physical symptoms at home** could improve your experience. What specifically could the clinic change **to help you**?

- ☐ Help with lack of energy or feeling generally unwell
- ☐ Help with pain, nausea or vomiting
- ☐ Advice on the medication you need to take at home
- ☐ Ensure prescription refills are available quickly
- ☐ Help you manage any weight loss or gain
- ☐ Help you cope with hair loss

On the last screen you indicated that help with **emotions and relationships at home** could improve your experience. What specifically could the clinic change **to help you**?

- ☐ Ensure you know about a range of available support services
- ☐ Provide better information about possible treatment outcomes
- ☐ Provide better information on changing or distressing emotions
- ☐ Provide support services near the clinic

SPECIFIC INITIATIVES: At home

On the last screen you indicated that help with **daily activities and lifestyles** could improve your experience. What specifically could the clinic change **to help you**?

- ☐ Services to help with household chores (like yard work, cleaning or cooking)
- ☐ Services to help with showering, bathing or dressing
- ☐ Medical aids or devices (like raised seats or hand rails)
- ☐ Mobility inside or outside your home
- ☐ Information about being active and having a healthy diet
- ☐ Help to reduce unhealthy activities (like smoking or drinking)

On the last screen you indicated that **help with work, finances or insurance** could improve your experience. What specifically could the clinic change **to help you**?

- ☐ Help with completing your sick leave forms
- ☐ Providing information about how long you may be away from work
- ☐ Advice and support about returning to work
- ☐ Help with coordinating insurance claims
- ☐ Finding information on available financial support

SPECIFIC INITIATIVES: At home

On the last screen you indicated that help **getting information at home** could improve your experience. What specifically could the clinic change **to help you**?

- ☐ Provide trustworthy information sources
- ☐ Knowing the long-term impacts of your condition or treatment
- ☐ Information about the changes to your daily life
- ☐ Being aware of possible symptoms
- ☐ Being aware of possible treatment side effects
- ☐ Knowing why specific tests are needed
- ☐ Knowing patient rights and responsibilities
- ☐ Information on remission, recurrence or spreading symptoms

On the last screen you indicated that **help involving family and friends** could improve your experience. What specifically could the clinic change **to help you**?

- ☐ Available professional counselors
- ☐ Help to discuss your condition and treatment with family and friends
- ☐ Family and friends being made comfortable within the clinic
- ☐ Involving family and friends in discussions and decisions
- ☐ Family and friends are able to contact the clinic on your behalf

SPECIFIC INITIATIVES: At home

On the last screen you indicated that **help with medical emergencies** could improve your experience. What specifically could the clinic change **to help you**?

- ☐ Knowing which symptoms are an emergency
- ☐ Providing information to family or caregivers on how to handle an emergency
- ☐ Knowing what to do if the clinic is closed
- ☐ Knowing whom to contact first

Prioritization: Pick 5

We would also like to know which changes would **most** improve your experience.

This screen is based on your previous answers.

Please select the **5** changes that would **improve your experience the most**:

- [x] Schedule convenient appointment times
- [x] Make it easy to contact someone at the clinic
- [x] Make it easy to get to and from your appointments
- [x] Car parking
- [x] Comfortable or pleasant waiting room
- [ ] Reduced time in the waiting room
- [ ] Having family or friends with you in the waiting room
- [ ] Provide more information
- [ ] Ensure all your concerns are addressed

Prioritization: Instructions for relative prioritization exercise

Now we would like you to prioritise your top changes.

You have a total of **100 points** to distribute across your top changes.

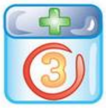

You can give points equally or give more points to those choices which are more important to you.

The more points you give to a change, the more important it is to you.

Touch where you would like to enter the points and the number pad will pop up on the screen.

You can see how many points you have left to distribute at the bottom of the screen in red.

Keep allocating points until you have 0 points left.

Prioritization: Point allocation

You have **100 points** to distribute. How many points would you like to give to:

Item	Points
Schedule convenient appointment times	50
Make it easy to contact someone at the clinic	10
Make it easy to get to and from your appointments	10
Car parking	25
Comfortable or pleasant waiting room	5

Total: 100
